# An improved method for diagnosis of Parkinson’s disease using deep learning models enhanced with metaheuristic algorithm

**DOI:** 10.1186/s12880-024-01335-z

**Published:** 2024-06-24

**Authors:** Babita Majhi, Aarti Kashyap, Siddhartha Suprasad Mohanty, Sujata Dash, Saurav Mallik, Aimin Li, Zhongming Zhao

**Affiliations:** 1 Department of CSIT, Central University, Guru Ghasidas Vishwavidyalaya, Bilaspur, Chhattisgarh 495009 India; 2 Department of Information Technology, Nagaland University, Dimapur, Nagaland India; 3 Department of Environmental Health, Harvard T. H. Chan School of Public Health, Boston, MA USA; 4 School of Computer Science and Engineering, Xi’an University of Technology, Xi’an 710048, China; 5 Center for Precision Health, School of Biomedical Informatics, The University of Texas Health Science Center at Houston, Houston, TX 77030 USA

**Keywords:** Parkinson’s disease, SPECT DaTscan, T1, T2-weighted, Deep learning, VGG16, InceptionV3, Grey wolf optimization

## Abstract

Parkinson's disease (PD) is challenging for clinicians to accurately diagnose in the early stages. Quantitative measures of brain health can be obtained safely and non-invasively using medical imaging techniques like magnetic resonance imaging (MRI) and single photon emission computed tomography (SPECT). For accurate diagnosis of PD, powerful machine learning and deep learning models as well as the effectiveness of medical imaging tools for assessing neurological health are required. This study proposes four deep learning models with a hybrid model for the early detection of PD. For the simulation study, two standard datasets are chosen. Further to improve the performance of the models, grey wolf optimization (GWO) is used to automatically fine-tune the hyperparameters of the models. The GWO-VGG16, GWO-DenseNet, GWO-DenseNet + LSTM, GWO-InceptionV3 and GWO-VGG16 + InceptionV3 are applied to the T1,T2-weighted and SPECT DaTscan datasets. All the models performed well and obtained near or above 99% accuracy. The highest accuracy of 99.94% and AUC of 99.99% is achieved by the hybrid model (GWO-VGG16 + InceptionV3) for T1,T2-weighted dataset and 100% accuracy and 99.92% AUC is recorded for GWO-VGG16 + InceptionV3 models using SPECT DaTscan dataset.

**Supplementary Information:**

The online version contains supplementary material available at 10.1186/s12880-024-01335-z.

## Introduction

Parkinson's disease, also known as neurodegeneration, is a long-term, neurological, and progressive motor illness [1] characterised by the progressive death of dopamine-producing brain cells. Dopamine is an organic substance produced by neurons that serves as a neurotransmitter in the brain, facilitating communication between neurons. Parkinson's disease results from impaired neuronal communication due to insufficient dopamine production in the brain. The substantia nigra, a small region where the neurons of the human brain are affected due to Parkinson’s disease.

A new United Nations research claims that nearly 1 billion people worldwide, or approximately one in six, suffer from neurological conditions like epilepsy, migraine, brain injuries, and neuro-infections like Alzheimer's, PD as well as stroke, and multiple sclerosis. Each year, 6.8 million of these sufferers pass away (https://news.un.org/en/story/2007/02/210312). Although the actual cause of Parkinson's disease is unknown, it is believed that a combination of inherited and environmental factors is responsible for it [2]. In the modern world, PD is affecting 2% to 3% of people who are at the age of 65 and older [3]. Parkinson's disease progresses differently in every patient, and it is impossible to anticipate how quickly the disease may progress in any specific person. While some people may have only minor symptoms for years, others may do so quite fast as they progress to more severe problems. Parkinson's disease often starts with minor tremors or other motor symptoms on one side of the body and progresses slowly over a number of years. The disease's symptoms could extend throughout the body and get worse, possibly affecting both sides of it. Even though Parkinson's disease is an ongoing and advancing condition, there are medicines that can help to manage symptoms and improve the standard of living. Parkinson's disease does not yet have an appropriate early diagnosis or treatment. Medication, physical therapy, and lifestyle modifications are some of its treatments. Parkinson's disease progression can be slowed down or stopped, even though there is presently no known cure for it.

These days, artificial intelligence (AI) approaches—machine learning (ML) and state of art deep learning (DL) are greatly assisting medical professionals in the early diagnosis of illnesses. Due to this, research has recently been done to automatically identify Parkinson’s disease using MRI images utilizing a variety of AI and ML algorithms. Many different diseases and ailments have been diagnosed using deep learning, and the findings frequently outperform traditional benchmarks [4].

Deep learning models have become powerful and are mostly used in image classification problems. With their ability to learn intricate patterns and features from images, they can often surpass traditional machine-learning approaches in accuracy. It automatically extracts relevant features from images, hence causing the elimination of manual feature engineering. This feature extraction capability permits the model to learn complicated representations and capture both low-level and high-level features present in the images. It can handle large-scale datasets efficiently. They can learn from vast amounts of labelled data, which is essential for training accurate image classifiers. Deep learning frameworks and libraries are designed to leverage parallel computing resources like GPUs to accelerate training and inference processes.

Over the past two decades, meta-heuristic optimization techniques have gained a lot of popularity. A few of these include particle swarm optimization (PSO) [5], grey wolf optimization (GWO) [6], ant colony optimization (ACO) [7], artificial bee colony optimization (ABC) [8], etc. Hyperparameter tuning is one of the tedious jobs to manually fine-tune the parameters to obtain the best optimal values. The population-based metaheuristic algorithm known as grey wolf optimization (GWO) is influenced by the way grey wolves hunt. It searches for the best answers in a problem area by combining exploration (diversification) with exploitation (intensification). It is used to automatically fine-tune the parameters which mimic the social behaviour of grey wolves, including their leadership hierarchy and group hunting. The improved capacity of GWO prevents results from being stuck in the local optimal value [9]. It also finds the best solution with a quick convergence rate.

The benefits of deep learning and GWO in image classification include higher accuracy, autonomous feature extraction, scalability, transfer learning skills, robustness to fluctuations, finding the optimal solutions, automatic hyperparameter tunning and continuous progress through continuing research and development. A variety of AI approaches using ML and DL models have been created in the past. In this study, a new framework is employed by combining grey wolf optimization (GWO) with four deep learning models known as VGG16 [10], DenseNet [11], InceptionV3 [12], DenseNet-LSTM [13] and a hybrid model VGG16 + InceptionV3.

The following is a concise explanation of the paper's main contribution.

(I) Number of images created empty tuples, from which the performance of deep learning models degrades. These empty tuples are removed to obtain better performance by using the Python function.
(II) Proposed four deep learning models with hyperparameter optimization by GWO known as GWO-VGG16, GWO-DenseNet, GWO-InceptionV3, and GWO-DenseNet-LSTM.
(III) Proposed hybrid model using GWO-VGG16 + InceptionV3.
(IV) The proposed models are compared with the existing models using various performance metrics.

Following is the format for the remaining section: The earlier studies are covered in “Related literature” section. “Materials and methods” section explains the preprocessing of MRI images and the development of methodologies. Experimental results and discussions, comparisons between the existing models and proposed models are discussed in “Results and discussion” section. In “Conclusion and future work” section, conclusions and future scope are discussed briefly.

## Related literature

In the past few years, various studies have been created and published by academics worldwide to help in Parkinson's disease diagnosis. Many of these researchers have used various AI methods to analyse and classify the MRI brain images in order to detect various diseases related to Parkinson’s disease. Deep learning techniques are the most often used method for classifying MRI images, due to their capacity to deliver superior results than those obtained by more conventional machine learning techniques. This particular section explains the research using ML and DL methods to diagnose patients with Parkinson’s disease.

### Related review literature using T1, T2-weighted dataset

Camacho et al., (2023) [14] have developed a robust explainable deep learning classifier trained for the classification of Parkinson’s disease using T1-weighted MRI dataset. A total of 1,024 PD and 1,017 subjects from matched controls (HC) of the same age and gender are gathered from 2,041 MRI data i.e. T1-weighted MRI datasets from 13 separate investigations. The datasets undergone a skull-stripping process, isotropic resampling, bias field correction, and nonlinear registration to the MNI PD25 atlas. Convolutional Neural Network is trained to categorise PD and HC participants using the Jacobian maps produced from the fields of deformation and fundamental clinical data. The authors provide improved knowledge of the clinical variables associated with Impulse Control Behaviors (ICB) and structural and functional brain abnormalities in PD patients. They have measured grey and white matter brain volume, and graph topological features using multimodal MRI data [15]. A new technique is introduced for categorising a person's 3-D magnetic resonance scans as a diagnostic tool for Parkinson's disease by using one of the largest Parkinson’s Progressive Marker Initiative (PPMI) MRI datasets from a patient group with the condition and healthy controls. Due to the fact that gender has a substantial impact on neurobiology and PD cases are developed more likely in males than women, it is advantageous that different research is conducted for men and women [16]. The viability and usefulness of employing multi-modal MRI datasets to automatically distinguish between PD, PSP-RS, and HC subjects are examined. For this investigation, there are 45 PD, 20 PSP-RS, and 38 HC subjects with available T1-weighted MRI datasets, T2-weighted MRI datasets, and diffusion-tensor (DTI) MRI datasets [17]. Brain morphology using T1-weighted, brain iron metabolism using T2-weighted, and microstructural integrity using DTI dataset regional values are determined by an atlas-based approach. These values are used to choose features, and then classification is performed using a variety of well-known machine learning approaches. A 3D CNN architecture is proposed after data pre-processing in order to learn the complex patterns in MRI images for the identification of Parkinson's Disease. From the baseline visit, 406 individuals including 203 in good health and 203 with Parkinson's disease are selected for the experiment [18].

A novel method is used which trains a deep neural network model using data from new patients, specifically with T1 MRI and DaTscan datasets. The information utilized to model the knowledge retrieved from the PPMI database contains a set of vectors that represent the clustering centers of these representations, along with the matching Deep Neural Network (DNN) structure. The ability of the unified model created using these many datasets to predict Parkinson's disease in an effective and transparent manner has then been demonstrated [19]. Two new deep learning techniques are proposed for ensemble learning-based Parkinson's disease detection. Instead of using the entire MRI image, authors focused on the Grey and White Matter areas which greatly improved detection accuracy and obtained 94.7% accuracy [20]. To discover which brain regions are important in the decision-making process for architecture is performed by occlusion analysis as well. Multiple parcellated brain areas are used to train a CNN. The idea is to create a complicated model by combining the models from various locations using a greedy algorithm. Three retrospective investigations included 305 PD patients (59.9–9.7 years of age) and 227 HC patients (61.0–7.4 years of age). Based on the Automatic Anatomic Labelling template, fractional anisotropy and mean diffusivity are determined and then divided into 90 different brain regions of interest (ROIs) [21].

The authors have suggested CNN with eight layers deep for 3D T1-weighted MRI images to differentiate between PD and HC individuals. The proposed model additionally made use of the information provided by the individuals' ages and genders. In addition, batch and group normalization are applied to the designed model, increasing the accuracy up to 100% [22]. An autonomous diagnosis approach that distinguishes the PD and HC with high accuracy. Benchmark T2-weighted MRI scans for both PD and HC are made available to the public by the PPMI. Image registration technique is used to choose and align the middle 500 slices of a T2-weighted MRI scan [23].

The study evaluates the viability of machine learning techniques for classifying patients with Parkinson's disease (PD) and non-proliferative osteoporosis (NPOD) using 30 patients data. It demonstrates that PD patients can be distinguished from NPOD patients by (a) using T1-weighted axial magnetic resonance imaging (MRI) scans, or (b) using morphometric measurements such as cortical thickness, cortical surface area, and volumetric measurements of the brain's subcortical and cortical areas division [24].

Based on the analysis performed using the brain MRI slices, authors [25] has suggested machine-learning-technique (MLT) to assess and classify the tumour locations into low/high grade using 30 patients. A series of operations, including pre, post and classification procedures, are carried out by MLT. The Social Group Optimization (SGO) method in conjunction with Fuzzy-Tsallis thresholding improves the tumour section during pre-processing. For the mining of the tumour area Level-Set Segmentation (LSS) is utilized in the post-processing step.

### Related review literature using SPECT DaTscan dataset

Thakur et al. (2022) [11] have constructed a CNN model that can accurately pinpoint the ROIs after feature extraction. In the study, 1,390 groups of DaTscan images with PD and normal classes are examined. The final classification layer includes a soft-attention block which makes use of the DenseNet-121 design. After classifying the images, Soft Attention Maps and feature map representation are reutilized to visually analyse the region of interest (ROI). The work sought to establish an ensemble deep learning technique with three stages for PD patient prognosis. Retrospective information on 198 Parkinson's disease (PD) patients is obtained from the PPMI database and then randomly 118 patients are assigned to training, and 40–40 patients are assigned to both validation and test sets. The features are extracted from the DaTscan dataset and clinical assessments of motor symptoms in steps 1 and 2. In step 3, an ensemble of DNN are trained to predict 4 years of patient outcome [26]. A CNN model is created that can distinguish between PD patients and HC patients based on SPECT images. In this study, 2723 images of the SPECT dataset are used out of which 1364 samples from the PD group and 1359 samples from the HC group. The image normalization method is used to improve the regions of interest (ROIs) required for the network to learn attributes that set them apart from other regions of interest (ROIs). In order to assess the effectiveness of the network model, tenfold cross validation is used [27]. Six well-known interpretation techniques and four deep-convolutional neural network are designed [28]. Also, the authors suggest a mechanism for evaluating interpretation performance as well as a way to use interpreted input to aid in model selection. It is suggested to develop a computer learning model that accurately identifies whether every given DaTscan has PD or not while offering a logical justification for the prediction. Visual indicators are created utilizing Local Interpretable Model-Agnostic Explainer (LIME) approaches. Further, transfer learning is used to train DaTscans on a CNN (VGG16) from the PPMI database, and the resulting models have 95.2% accuracy. Finally, the paper concludes that the suggested approach may successfully assist medical professionals in PD detection because of its measured interpretability and accuracy^11^. To analyse pictures from dopamine transporter single-photon emission computed tomography (DAT-SPECT) has been suggested utilizing an ANN. With the use of an active contour model, striatal regions are segmented and utilized as the data performing transfer learning on the artificial neural network which is pre-trained to distinguish Parkinson’s disease. To serve as a benchmark, the support vector machine is trained to use semi-quantitative measurement metrics including the specific binding ratio (SBR) and asymmetry index [29].

The active contour model is utilized to segment the striatal regions in the images. These segmented regions are then employed as the dataset for an already-trained ANN to do transfer learning. The goal is to separate PD from Parkinsonism associated with other diseases. Artificial neural networks (ANN) and image processing techniques are proposed to identify Parkinson's disease in its early stages [30]. The images used are 200 SPECT scans from the PPMI dataset, out of which 130 are of normal participants and 70 are of Parkinson's disease (PD) patients. Using the sequential grass fire algorithm, the caudate and putamen areas of the images are determined. To distinguish healthy and Parkinson's disease-infected people, these above features are loaded into an ANN. A novel approach is introduced for the medical treatment of neurodegenerative disorders, like Parkinson's, that utilizes trained DNNs to extract and utilize latent information. The paper uses transfer learning along with k-means clustering, K-NN classification, and DNN trained representations to enhance disease prediction using MRI data [31]. In the recent past, authors have presented a model for the early identification of PD which combines image processing with ANN in order to improve the imaging diagnosis of PD. The caudate and putamen serve as the study's region of interest (ROI), and the model identified them by analysing 200 SPECT images from the PPMI database, out of which 100 are of healthy people and 100 are of PD people. The ANN is then fed with the ROI area data, with a thought it will recognise patterns similar to how a human observer would do [32]. A novel method is suggested that uses 3-dimensional convolutional neural networks (CNNs) to differentiate between PD and healthy control. In order to reduce overfitting and boost the neural network's generalisation abilities, the training set as well as the data from this set's sagittal plane using a straightforward data augmentation technique is given as input to the model [33].

One of the difficult challenges all are facing is determining Parkinson's disease in the early stages. To conduct research on the early detection of PD using MRI images, various authors developed numerous computer-based machine learning and deep learning methods as described above.

In this study, authors have proposed four deep learning models whose hyperparameters are optimized using GWO, namely GWO-VGG16, GWO-DenseNet, GWO-DenseNet + LSTM, GWO-IncepionV3, and a hybrid model (GWO-VGG16 + InceptionV3) which is the novelty of this paper. No authors earlier used these models with T1, T2-weighted and SPECT DaTscan for PD detection. Here, a number of images are creating empty tuples, from which the performance of the deep learning models degrades. These empty tuples are properly handled and removed to obtain better performance. This problem has also never been addressed by any authors previously in the literature.

## Materials and methods

**Fig. 1:**
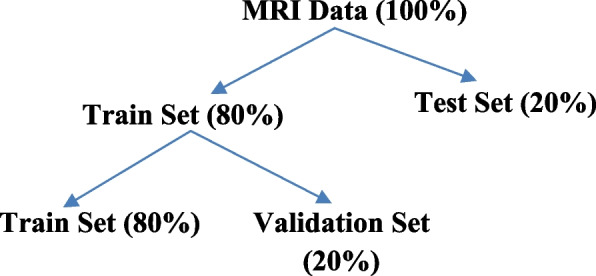
Splitting of MRI datasets

This section illustrates the proposed methodology, preprocessing of MRI images, and model development. After that, the data is divided into two sets using an 80:20 ratio for the train and test sets. Again, the train set is divided into train and validation sets. The 80% of input images are fed to the proposed model for training and then the models are validated using 20% from the train set samples. Finally, models are tested using the remaining 20% of the data. The distribution is depicted in Fig. 1 below.

### Proposed methodology

**Fig. 2:**
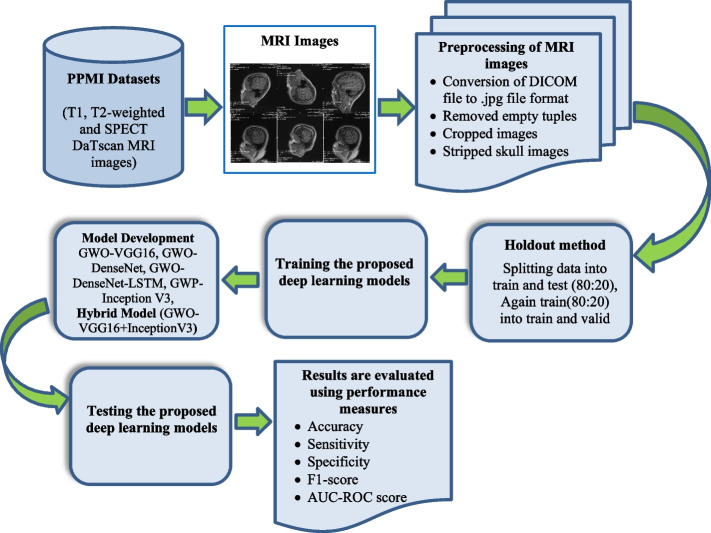
Proposed methodology for early detection of PD using GWO and deep learning models

In the proposed methodology, steps are given in the following:

- Step 1: Firstly, T1, T2-weighted and SPECT DaTscan MRI datasets are collected from the PPMI website.
- Step 2: MRI images are then pre-processed using preprocessing techniques such as the conversion of DICOM file to.jpg format, cropped images using MicroDICOM Viewer desktop application, removing empty tuples and finally skull stripping is done using the python package (simple ITK). Normalization is also done using batch normalization for scaling.
- Step 3: Datasets are divided into train and test sets using the holdout method (80:20 ratio) Again train set is divided into (80:20 ratio) two sets i.e. train and validation set.
- Step 4: Four deep learning models are proposed whose hyperparameters are optimized by GWO, known as GWO-VGG16, GWO-DenseNet, GWO-DenseNet-LSTM, GWO-InceptionV3, with one hybrid model GWO-VGG16 + InceptionV3.
- Step 5: Finally, results are evaluated using various performance measures such as accuracy (acc), sensitivity (sen), specificity (spe), precision (pre), f1_score (f1-scr) and AUC score. The proposed methodology is also graphically presented in Fig. 2

### MRI data collection

The MRI data are extracted from PPMI website [34]. The PPMI dataset is a large-scale longitudinal investigation of Parkinson's Disease (PD) conducted by the Michael J. Fox Foundation for the research of Parkinson's. The objective of the study is to find biomarkers that can aid in predicting the onset and progression of PD and to create new treatments for the condition. The PPMI dataset contains a variety of information, including clinical evaluations, genetic information, biospecimen samples (blood and CSF), and brain imaging data (MRI and DaTscan). Researchers from all across the world can analyse and do research on the dataset.

One of the distinguishing characteristics of the PPMI dataset is its longitudinal nature, which monitors patients over a number of years. This feature enables researchers to examine changes in disease development and find potential biomarkers for the illness. The dataset also includes a large control group of healthy individuals, which provides a baseline for comparison. T1, T2-weighted MRI [35] and SPECT DaTscan [34] datasets used in this study are collected from the PPMI website.

### MRI data samples

T1,T2-weighted and SPECT DaTscan dataset from PPMI are chosen for this investigation. A 1.5—3 Tesla scanner was used to create these pictures. The entire scan takes about twenty to thirty minutes. Three distinct views—axial, sagittal, and coronal—were used to acquire the T1, T2-weighted MRI images as a three-dimensional sequence with a slice thickness of 1.5 mm or less. The description of MRI images of both datasets is given below in Tables 1 and 2.

**Table 1:** Description of MRI images of T1,T2-weighted dataset

Dataset	Dimension	Flip Angle	Thickness of slice	Slice orientation	Matrix original size	After pre-processing matrix size	Voxel Size
T1,T2-weighted MRI image	3D	9 degrees	1.5 mm	Sagittal	256 × 256	224 × 224	1 × 1 × 1 mm^3^

**Table 2:** Description of MRI images of SPECT DaTscan dataset

Imaging mode	Orbit	Matrix	Acquisition Zoom	Angle	Time	No. of Projections	Slice orientation	Radius
Step and Shoot	Circular	128 × 128	1.23	3 degrees	30 s	60 projections	Sagittal	15 cm

In this study, two datasets are used i.e. T1,T2-weighted and SPECT DaTscan. A total of 30 subjects are included in T1,T2-weighted MRI dataset from which 15 subjects (Male-7, Female-8) are Parkinson’s disease (PD) and 15 subjects (Male-7, Female-8) are healthy control (HC) which contains a total number of 9070 MRI images of different sizes. Out of 9070 MRI images, 3620 are PD subjects and 5450 are HC subjects. A total of 36 subjects are included in SPECT DaTscan dataset from which 18 subjects (Male-9, Female-9) are suffering from Parkinson’s disease (PD) and 18 subjects (Male-9, Female-9) are healthy control (HC) which contains a total of 20,096 MRI images. Out of 20,096 MRI images, 5752 are PD subjects, and 14,344 are HC subjects. The sample size is distributed as shown in Table 3.

**Table 3:** MRI data sample distribution

Datasets used	Total no. of subjects		Subject Division (80%:20%)	Total no. of image samples used		Training samples (80%)		Testing (20%)	
						**Training samples (80%)**	**Validation samples (20%)**		
T1, T2-weighted MRI images	**30**		**24:6**	**9070**		**7256** (PD-2896, HC-4351)		**1814**	
	PD	15		PD	3620	5805	1451	PD	724
	HC	15		HC	5450			HC	1090
SPECT DaTscan	**36**		**29:7**	**20096**		**16077** (PD-4601, HC-11475)		**4019**	
	PD	18		PD	5752	12,862	3215	PD	1151
	HC	18		HC	14344			HC	2869

#### Inclusion criteria

Those patients are included in the study whose age is between 55 and 75 years. Only PD and HC subjects are included.

#### Exclusion criteria

Patients whose age is less than 55 and greater than 75 are excluded from this study. Other category subjects are excluded, such as SWEDD, PRODROMAL, etc.

### Image pre-processing

MRI images are available in DICOM (Digital Imaging and Communications in Medicine) (https://www.microdicom.com/dicom-viewer-user-manual/) file format which is used to store and send medical pictures like X-rays, CT scans, and MRIs. A lot of image-related metadata, including patient data, information on the image's acquisition, and other medical data, is included in DICOM files. However, the DICOM file format is difficult to deal with when employing these pictures for machine learning tasks.

Many machine learning libraries and frameworks don't natively support DICOM files, which is one of the reasons DICOM images are generally transformed to other image formats, like png or jpg, before being used for image classification. Although Python has libraries for reading and manipulating DICOM files, it can often be simpler to convert the images to a more widely used format, such as png or jpg, and then use conventional image processing packages to work with the images.

Another reason for converting DICOM images to jpg is that DICOM images have different pixel representations and bit depths, depending on the specific equipment and software used to generate them. Jpg images, on the other hand, have a standardized pixel representation and bit depth, making them more consistent and easier to work with.

Finally, unlike some other picture formats, png, jpg images don't lose any information when they are compressed, which might be crucial in the area of medical imaging, where even minor data loss can have serious repercussions.

**Fig. 3:**
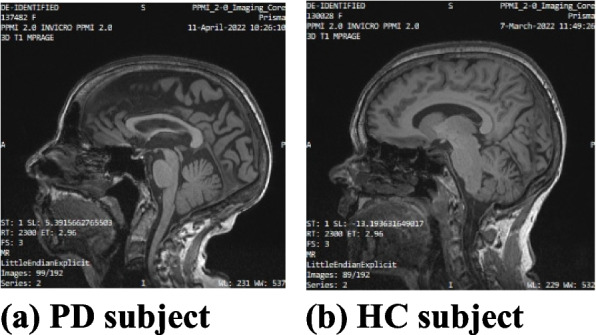
Original MRI brain images of T1, T2-weighted dataset (**a**) PD subject (**b**) HC subject

In this study, all the DICOM (.dic) file format images are first converted into the .jpg format using MicroDICOM Viewer desktop application. The original image size is 256 × 256 × 3. The images which generate empty tuples are removed from the selected images. Empty tuples are those that create the null arrays for which the machine learning models create a huge number of misclassifications. These images are removed based on the threshold value of 30 pixels. Then images are cropped and stripped using Python library functions. Then, images are normalized using batch normalization. After preprocessing, the final size of the MRI images is 224 × 224 × 3, which is given as input to the models. The original MRI images are shown in Fig. 3a and b.

**Fig. 4:**
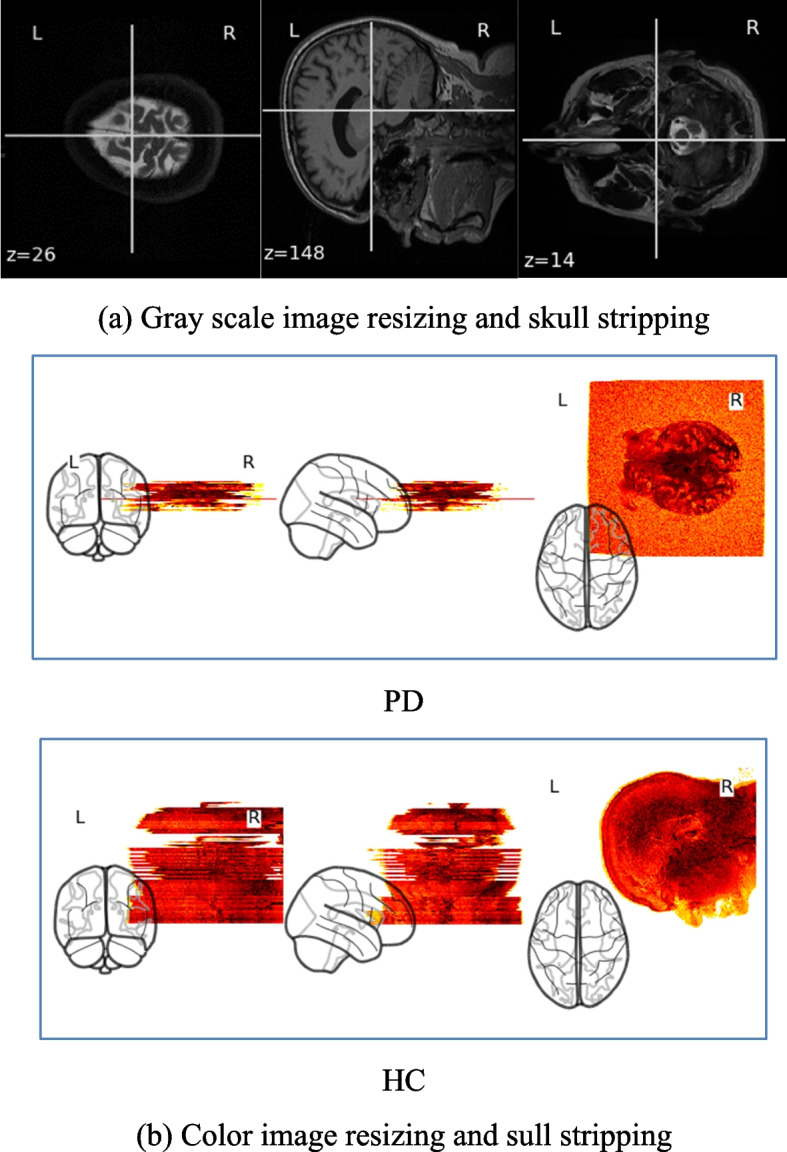
Image resizing and skull stripping of T1,T2-weighted dataset of both the PD and HC subjects in (**a**) gray scale and in (**b**) color

After pre-processing the images are shown in Fig. 4a and b.

### Model development

Four deep learning models with the combination of grey wolf optimization technique GWO-VGG16, GWO-DenseNet, GWO-DenseNet-LSTM, GWO-InceptionV3 and a hybrid model GWO-VGG16 + InceptionV3 have been proposed in this study for detection of PD accurately. All the proposed models are explained briefly below:

- **VGG16:** VGG16 (Visual Geometry Group 16) [10] is a deep CNN architecture that was suggested by the University of Oxford's Visual Geometry Group in 2014. It is created for image classification problems and has accomplished state-of-the-art performance on various benchmarks, including the ImageNet Large Scale Visual Recognition Challenge (ILSVRC) dataset. Thirteen Conv (convolutional) layers, 3 fully connected dense layers, and other layers made up the 16-layer, VGG16. The input layer accepts an image as input of size 224 × 224 × 3. Each of the 13 convolutional layers is having 3 × 3 filters with a stride of (1). After each max pooling layer, the number of filters doubles i.e. 6 × 6 with a stride of 2, starting with the first convolutional layer that includes 64 filters. The max pooling layers help to decrease the number of model parameters and avoid overfitting by reducing the spatial dimensions of the output by a factor of 2. Padding is a technique that is used by all convolutional layers to guarantee that the output's spatial dimensions match those of the inputs. Rectified linear unit (ReLU) is one of the activation functions that introduces nonlinearity into the model comes after each convolutional layer. It has 2 fully connected layers, each with 256, and 128 neurons respectively. There are 128 neurons in the output layer, corresponding to the two classes in the T1,T2-weighted and SPECT DaTscan datasets. In order to output a VGG16 algorithm is renowned for its ease of use and capacity to extract intricate information from images. However, it can be expensive to train and utilize computationally probability distribution over the classes, it uses a “sigmoid” activation function. VGG16 is a very deep network with huge parameters.
- **DenseNet:** DenseNet [11], short for Dense CNN, is a deep learning architecture that Huang et al. have first presented in 2016. It is designed to address the vanishing gradient problem and encourage deep neural networks that reuse features. It creates connections that are dense between all layers. Each layer in this architecture receives feature maps from all levels below it as input. Gradient flow throughout the network is made possible by this connection structure, which provides direct access to features at various depths.

DenseNet is made up of dense blocks, each of which has several levels. Each layer in a dense block is connected to all layers before it. The overall network design is created by gradually connecting dense units. Convolutional and pooling layers are employed as transition layers to shorten the distance between packed blocks. They contribute to preserving connections while lowering computational complexity and feature map sizes. The key advantages of DenseNet are feature reuse, parameter efficiency, and mitigating the vanishing gradient problem.

DenseNet is widely used and has produced state of art outcomes for a number of computer vision applications, such as semantic segmentation, image classification and object recognition. It is now a well-liked option among deep learning researchers and practitioners.

- **DenseNet-LSTM:** DenseNet with LSTM [13] refers to a network that combines Long Short-Term Memory (LSTM) networks with the DenseNet network. The strengths of LSTM's modelling of sequential data and ability to detect temporal relationships are combined with DenseNet's feature extraction skills in this hybrid architecture.

The DenseNet component serves as the feature extraction backbone. The dense connections and hierarchical structure aid in the efficient acquisition of both local and global image features. At various degrees of abstraction, the DenseNet layers process the input image or sequence to extract significant information.

Afterwards, an LSTM network receives the output from the DenseNet layers. The LSTM is a form of recurrent neural network (RNN) that excels at modelling sequential data because it preserves long-term dependencies and detects temporal patterns. Memory cells are present in the network, allowing it to selectively recall or forget information over time. DenseNet and LSTM are used in various applications such as video action recognition, natural language processing, sentiment analysis, etc. in order to identify actions or activities, DenseNet captures features from individual frames, while LSTM processes the sequence of features.

- **InceptionV3:** InceptionV3 [13] is a variant of the Inception architecture that is introduced by Christian Szegedy et al. in 2015. InceptionV3 is a deep neural network that is created for image classification and object detection tasks. It consists of an input layer, stem network, inception modules, auxiliary classifiers, average pooling, fully connected dense layers and a final (output) layer.

The images are given as input to the input layer, typically of size 224 × 224 × 3. The stem network extracts feature from the input images using three convolutional layers. With a 3 × 3 kernel, the first, second and third layers consist of 32, 32 and 64 filters respectively. The max pooling layer, which follows the stem network, has a 3 × 3 filter with a stride of 2.

There are several inception modules in InceptionV3 that are responsible for doing feature extraction at various scales. Each inception module is made up of a number of convolutional layers with pooling layers and of various filters of sizes (1 × 1, 3 × 3, and 5 × 5) concatenated along the channel dimension. Compared to conventional convolutional layers, Inception modules are computationally inexpensive. Two auxiliary classifiers are included in InceptionV3 after the 5^th^ and 9^th^ inception modules. The auxiliary classifiers are made up of a dropout layer, a softmax activation function, a ReLU activation function, a fully connected layer with 1024 neurons, and a global average pooling layer. The auxiliary classifiers' role includes supplying the network with more training data and minimizing the vanishing gradient issue.

After the last inception module, InceptionV3 utilizes a global average pooling layer to shrink the output's spatial dimensions to a 1 × 1 feature map. A fully connected layer with 128 neurons is fed with the output of the global average pooling layer, which corresponds to the two classes in the T1,T2-weighted and SPECT DaTscan datasets. The fully connected layer outputs a probability distribution over the classes using a sigmoid activation function.

#### Proposed hybrid model (VGG16 + InceptionV3)

**Fig. 5:**
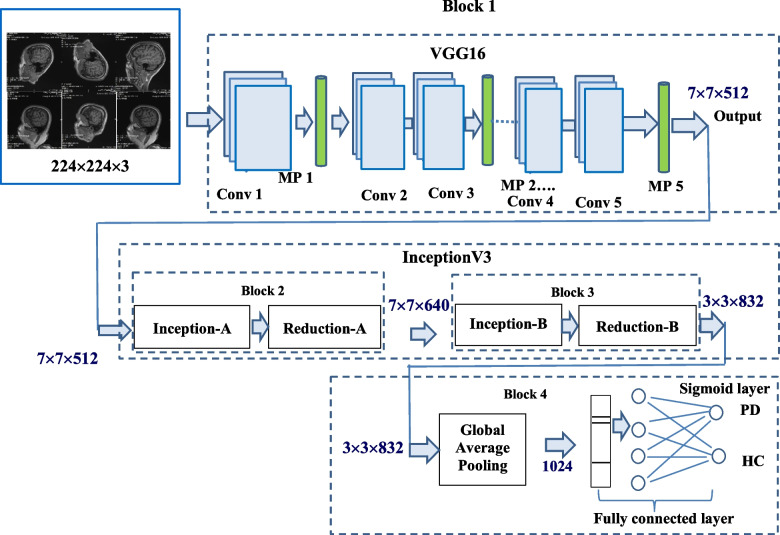
Architecture of the proposed Hybrid Model (VGG16 + InceptionV3) for PD detection

The proposed hybrid model is the fusion of VGG16 [10] and two blocks of InceptionV3 [36] as illustrated in Fig. 5. All sixteen layers of VGG16 makes the Block-1 followed by two blocks Block-2 and Block-3 of inception-reduction. Then in Block-4 global average pooling, fully connected and sigmoid layers are used. The size of the input to the Block-1 (VGG16) is 224 × 224 × 3 and output matrix size from it is 7 × 7 × 512. This output is passed as input to the inception-reduction block (named as Block-2) and output matrix obtained is of the size 7 × 7 × 640. Same process is repeated in Block-3 and size of the output is 3 × 3 × 832. Finally, in Block-4, global average pooling is executed and its output is fed to the fully connected layer to detect the patient as PD or HC.

**Fig. 6:**
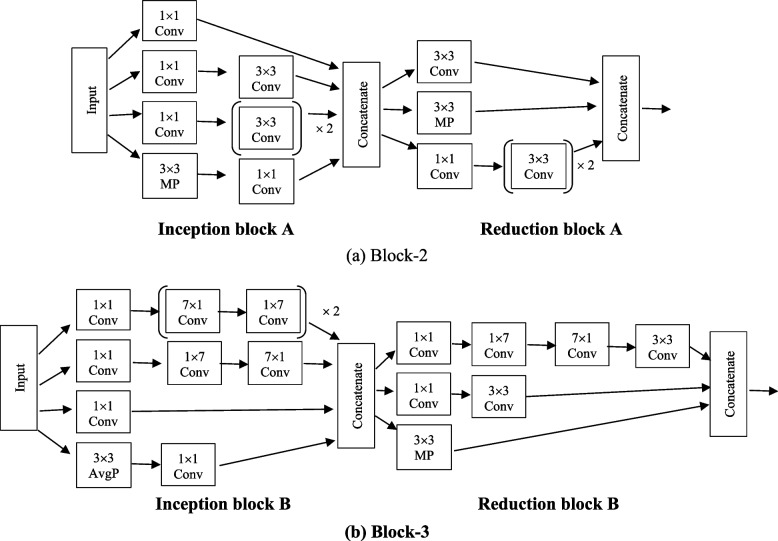
Architecture of (**a**) Block-2 and (**b**) Block-3 of Inception V3 models

Figure 6a and b present the detailed structure of Block-2 and Block-3 which are made up of inception-reduction blocks. Four (1 × 1) convolution, three (3 × 3) convolution, and maxpooling of kernel size (3 × 3) are present in the inception block. In comparison to the (3 × 3) convolution, the (1 × 1) convolution has a smaller coefficient which can decrease the number of input channels, and speed up the training process [37]. To extract the image's low-level features, such as edges, lines and corners, the (1 × 1) and (3 × 3) convolution layers are used. These are concatenated and routed to the reduction block. To prevent a representational blockage, the reduction block is employed which is made up of three (3 × 3) convolution, one (1 × 1) convolution and maxpooling layers. The advantage of using this Block-2 is that it lowers the cost and increases the efficiency of the network. The Block-3 is exhibited in Fig. 6b, which is also contains one inception and one reduction blocks. The generated output from the Block-2 is passed as input to the Block-3. To extract high-level features like events and objects, a convolution with a kernel of size (7 × 7) is employed. In place of (7 × 7) convolution, a (7 × 1) and (1 × 7) convolutions are used. The inception block consists of four (1 × 1) convolution and three sets of [(7 × 1) and (1 × 7)], as well as (3 × 3) average pooling layers [36]. Comparing the model to a single (7 × 7) convolution, factorization reduces the model's cost. Afterwards, the reduction block receives all of these layers concatenated together. The reduction block consists of two (1 × 1) convolution, two (3 × 3) convolution, one set of [(7 × 1) and (1 × 7)] and one (3 × 3) maxpooling layer. The output from the Block-3 is passed as input to the Block-4, global average pooling layer which determines the image's overall feature average. After that, the output of global average pooling layer is passed to the fully connected layer. The detailed output of each convolution layer is presented in Table 4. Finally, the predicted class is determined by selecting the class with the highest probability, which is represented by the Sigmoid layer.

**Table 4:** The detailed output of each convolution layer of proposed hybrid model

Phase	Feature Maps
Block-1 (VGG16)	7 × 7 × 512
Block-2 (Inception-reduction)	7 × 7 × 640
Block-3 (Inception-reduction)	3 × 3 × 832
Block-4 (Global Average Pooling Layer)	1024
Fully Connected Layer	1024
Sigmoid	1

The detailed output size of each layer is given below in Table 4.

#### Grey Wolf Optimization (GWO)

Seyedali Mirjalili introduced GWO in 2014 by imitating the social conduct, hierarchy of leadership, and hunting on the communal land of grey wolves [6]. Canidae is the family that includes the grey wolf (Canis lupus). As the top predators in the food chain, grey wolves are known as apex predators. The majority of grey wolves prefer to live in packs. The typical size of the group is between 5 and 12 people. Alpha, Beta, Delta and Omega are four different species denoted by (α), (β), (δ) and (ω).

The step-by-step procedure of grey wolf hunting is.

1. tracking, chasing, and approaching the prey.
2. As soon as the target starts moving, it is pursued, hounded, and surrounded.
3. attacking the prey or assaulting it.

In this section, social hierarchy, encircling, and attacking is mathematically represented as follows

- **Social hierarchy:** Alpha is the best solution (α) to mathematically express the social hierarchy, followed by (β) and (δ) as the next two best options. The remaining candidate solution is the (ω). α, β, and δ serve as the hunting (or optimization) cues in the GWO algorithm. The remaining ω wolves come after these α, β, and δ wolves.
- **Encircling/Surrounding Prey:** Grey wolves circle their prey during hunting. The encircling behavior is mathematically represented as

1 $$V=\left|\overrightarrow{S}.{\overrightarrow{T}}_{x}\left(t\right)-\overrightarrow{T}(t)\right|$$

2 $$\overrightarrow{T}\left(t+1\right)={\overrightarrow{T}}_{x}\left(t\right)-\overrightarrow{U}.\overrightarrow{V}$$

where the current iteration is denoted by t, coefficient vectors are denoted by S and U, the position of the prey is denoted by T_x_, and the grey wolf's position is denoted by T. The vector $\overrightarrow{S}$ and $\overrightarrow{U}$ are represented as

3 $$\overrightarrow{U}=2\overrightarrow{p}.\overrightarrow{{q}_{1}}-\overrightarrow{p}$$

4 $$\overrightarrow{S}= 2.\overrightarrow{{q}_{2}}$$

where q_1_, q_2_ are arbitrary vectors with a range of [0, 1] and the components of $\overrightarrow{p}$ decrease linearly from the value 2 to 0 throughout the course of iterations.

- **Hunting the prey:** Grey wolves have the ability to track down and encircle their prey. Typically, the alpha leads the hunt. Hunting may occasionally be done by the beta and delta. It is assumed that the most promising candidate solution, alpha, delta, improve knowledge of the potential prey’s location in order to mathematically replicate the hunting behaviour of the wolves. The three best candidate solutions are mathematically represented to update their position as follows –

Alpha Wolf, Beta Wolf, Delta Wolf

5 $$\overrightarrow{{V}_{\alpha }}=\left|\overrightarrow{{S}_{1}}.\overrightarrow{{T}_{\alpha }}-\overrightarrow{T}\right|$$

6 $$\overrightarrow{{V}_{\beta }}=\left|\overrightarrow{{S}_{2}}.\overrightarrow{{T}_{\beta }}-\overrightarrow{T}\right|$$

7 $$\overrightarrow{{V}_{\delta }}=\left|\overrightarrow{{S}_{3}}.\overrightarrow{{T}_{\delta }}-\overrightarrow{T}\right|$$

8 $$\overrightarrow{{T}_{1}}=\overrightarrow{{T}_{\alpha }}-\overrightarrow{{U}_{1}}.\left(\overrightarrow{{V}_{\alpha }}\right)$$

9 $$\overrightarrow{{T}_{2}}=\overrightarrow{{T}_{\beta }}-\overrightarrow{{U}_{2}}.\left(\overrightarrow{{V}_{\beta }}\right)$$

10 $$\overrightarrow{{T}_{3}}=\overrightarrow{{T}_{\delta }}-\overrightarrow{{U}_{3}}.\left(\overrightarrow{{V}_{\delta }}\right)$$

11 $$\overrightarrow{T}(t+1)=\frac{\overrightarrow{{T}_{1}}+\overrightarrow{{T}_{2}}+\overrightarrow{{T}_{3}}}{3}$$

The wolves in motion attack the prey when it stops. $\overrightarrow{U}$ is a randomly chosen number between -2r and 2r, while r_2_ is a number between -1 and 1. The search agent's next position is a position that falls somewhere between the object's most recent location and its preyer position. Thus, the attacking state is appropriate when $\left|\overrightarrow{U}\right|<1$ [38]. The behaviour of wolves is used to depict the process of finding the best solution. Following is the pseudocode of grey wolf optimization-

**Figure:**
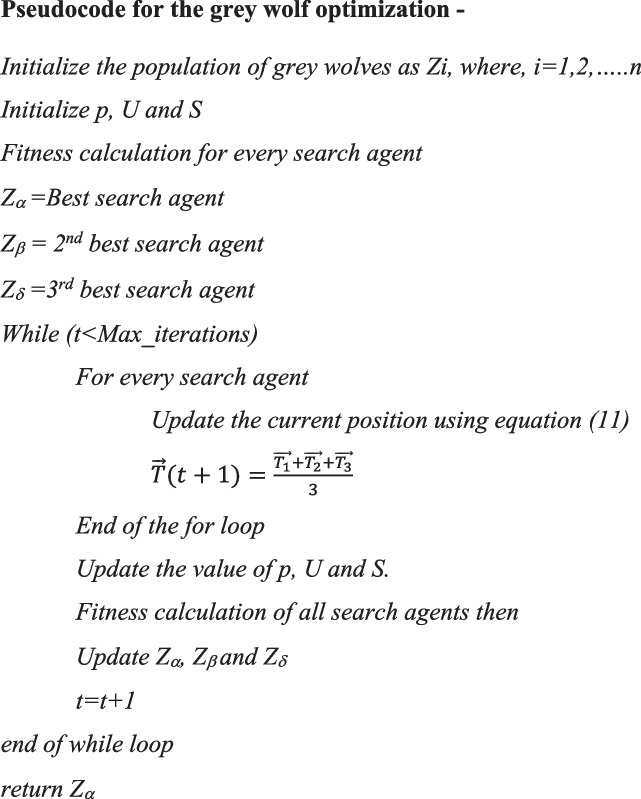
**Algorithm 1. **Hyperparameters optimization of deep learning models using GWO –

Step-by-step procedure:

- Step-1: Set the ranges of hyperparameter values. The ranges are given in Table 6.
- Step-2: Set the population size of the grey wolves.
- Step-3: Create an objective function that measures how well the deep learning models performed after being trained with the provided hyperparameters. This function gauges the model's effectiveness on a validation set.
- Step-4: Using the wolves' fitness levels, the dominance and hierarchy are determined.
- Step-5: Update the alpha, beta, delta and omega wolf's position using the Eq. (11)
- Step-6: Restrict the wolf’s new positions to remain inside each hyperparameter's stated ranges. A location is modified if it exceeds the limits.
- Step-7: Verify that the termination condition-such as completing the required number of iterations or obtaining the target fitness value-is met. The optimization procedure ends if the condition is satisfied; otherwise, return to step 5.
- Step-8: Take the optimal collection of hyperparameters for the deep learning models, which corresponds to the solution that is best and represents the wolf with the highest fitness value.

## Results and discussion

Prior to discussing the outcome, the fundamental performance evaluation criteria that are frequently used to evaluate different machine learning models while they are still in the training phase as well as in the testing phase are discussed in this section.

### Performance evaluation with confusion matrix

The confusion matrix [39] which is a two-dimensional table is used to determine performance metrics. It displays the actual and predicted class values which are represented by its elements as true positive (T + ve), true negative (T-ve), false positive (F + ve), and false negative (F-ve). The classification performance can be quantified using these four elements. Based on the confusion matrix, the five score metrics used in this study are as follows -

12 $$\mathrm{Accuracy}\;(\mathrm{Acc})\;=\frac{TP+TN}{TP+TN+FP+FN}$$

13 $$\mathrm{Sensitivity}\;(\mathrm{Se})\;=\;\frac{TP}{TP+FN}$$

14 $$\mathrm{Specificity}\;(\mathrm{Spe})\;=\frac{TN}{TN+FP}$$

15 $$\mathrm{Precision}\;(\mathrm P)\;=\;\frac{TP}{TP+FP}$$

16 $$\mathrm F1-\mathrm{score}=2\times\left(\frac{Precision\times Recall}{Precision+Recall}\right)$$

Along with the true + ve rate and false + ve rate, the ROC curve is shown on a graph which is known as the receiver operator characteristic. An area under the curve (AUC) score, or area under the curve, is also obtained.

### Experimental results analysis of all the proposed models

Experimental results are described by the following subsections that display the training results by plotting the accuracy and loss curves for each model used. For each model, the confusion matrix is also created and displayed.

#### Training results of all the proposed deep learning models

The experiments are carried out in Python by using various packages such as keras, opencv, tensorflow 2.1, scikitlearn [40] using the system configuration of intel Core i5 processor, 8^th^ Generation, with 16 GB RAM, and NVIDIA GEFORCE graphics combined with 8 GB memory. The standard T1, T2-weighted and SPECT DaTscan datasets are used for the study. The datasets are split into two sets i.e. train and test using an 80:20 ratio. Again, the training set is then split into train and validation sets. To train all the proposed deep learning models with GWO using the algorithm given in “Image pre-processing” section, various hyperparameters used are shown in Table 5 and the optimized hyperparameters by GWO are shown in Table 6.

**Table 5:** Hyperparameters used in all the proposed models

Models	Hyperparameters	Values
GWO-VGG16 GWO-DenseNet GWO-DenseNet-LSTM GWO-InceptionV3 Hybrid (GWO-VGG16 + InceptionV3)	No. of hidden layers	2
	Dropout	0.20
	Activation Function	ReLu
	Output Layer	Sigmoid
	Optimizer	Adam
	Loss Function	binary_crossentropy

**Table 6:** Optimized hyperparameters using GWO

Models	Manually tunning (range)	Learning rate	Batch-size	Momentum	Dense-units	epochs
GWO-VGG16	Lr = [0.001,0.01] Batch_size = [32, 128] Momentum = [0.9, 0.99] Dense_units = [128, 512] Epochs = [10, 50]	0.001	128	0.9	256, 128	30
GWO-DenseNet		0.001	128	0.9	256, 128	34
GWO-DenseNet-LSTM		0.01	128	0.95	256, 128	30
GWO-InceptionV3		0.001	128	0.9	256, 128	32
GWO-VGG16 + InceptionV3		0.01	128	0.99	256, 128	35

All the proposed deep learning models GWO-VGG16, GWO-DenseNet, GWO-DenseNet-LSTM, GWO-InceptionV3 and hybrid model GWO-VGG16 + InceptionV3 are pre-trained using the above hyperparameters. All the models comprise of an input layer, two hidden layers and an output layer. Every model has its own layers, such as convolutional, maxpooling, stem, global average pooling etc. Each layer consists of 256, and 128 neurons respectively. Every hidden layer ends with a dropout layer with 20 percent of neurons dropping out to overcome the overfitting problem. ReLu activation function is employed to all the hidden layers. To train all the models ‘adam’ optimizer and loss function ‘binary_crossentropy’ is used. The GWO algorithm is used for hyperparameter optimization with all the proposed models to obtain better performance. The ranges of parameters are given manually and optimized hyperparameters are shown in Table 6.

#### Testing results of all the proposed deep learning models

**Fig. 7:**
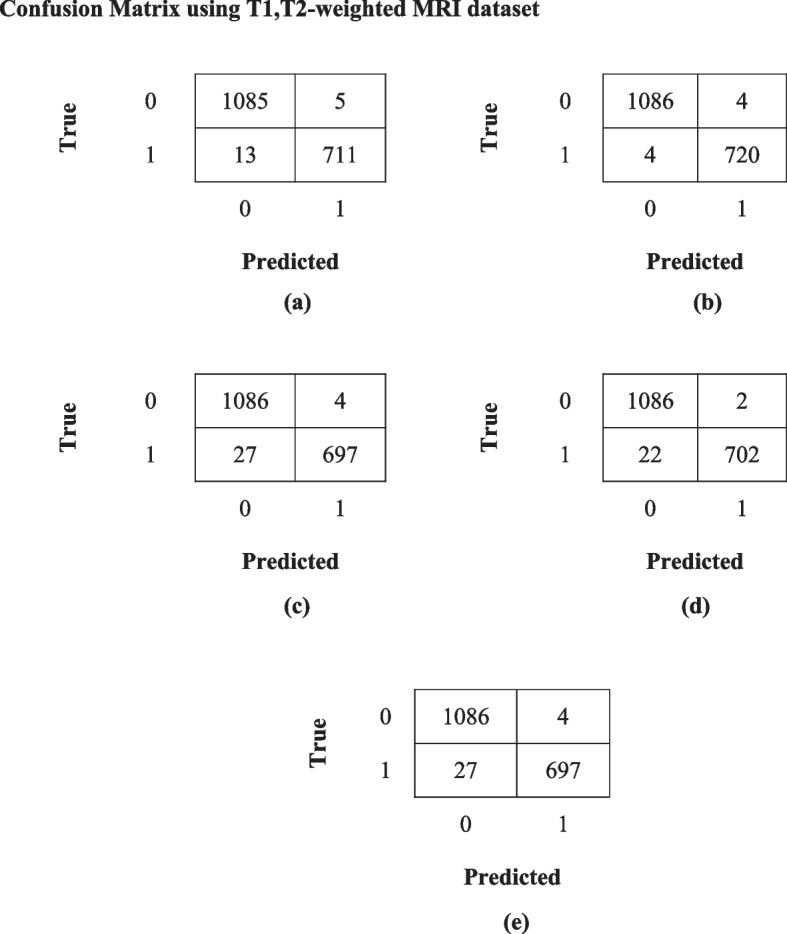
Confusion matrix for all the proposed models (**a**) GWO-VGG16, (**b**) GWO-DenseNet, (**c**) GWO-DenseNet-LSTM, (**d**) GWO-InceptionV3 and (**e**) Hybrid model (GWO-VGG16 + InceptionV3) using T1,T2-weighted MRI dataset

**Fig. 8:**
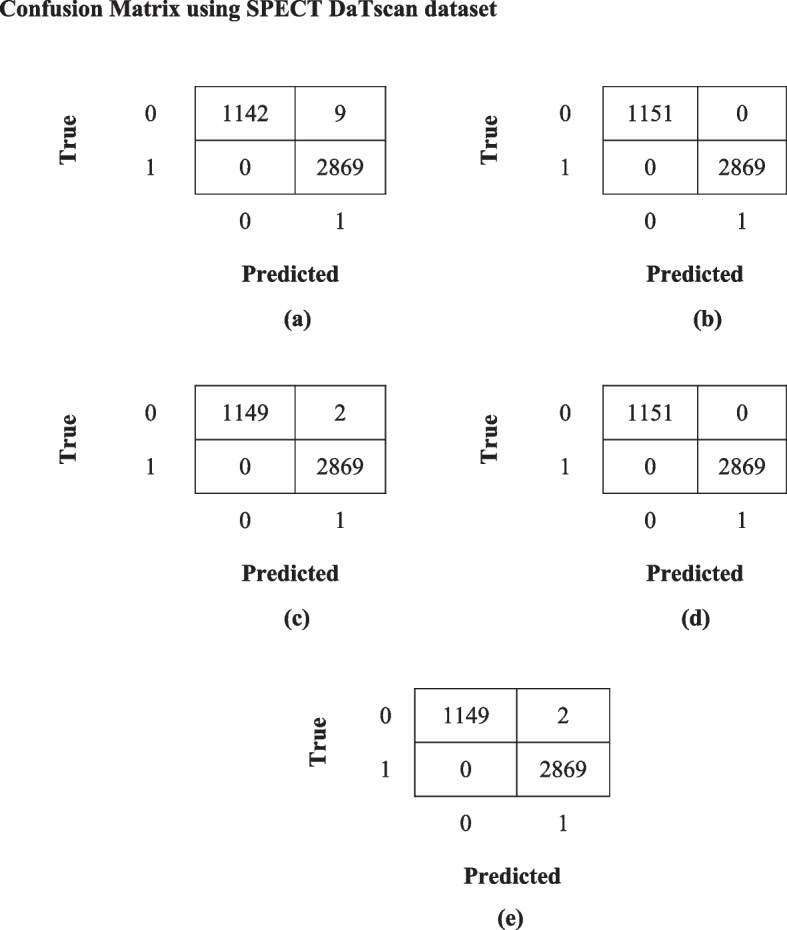
Confusion matrix of all the proposed models (**a**) GWO-VGG16, (**b**) GWO-DenseNet, (**c**) GWO-DenseNet-LSTM, (**d**) GWO-InceptionV3 and (**e**) Hybrid model (GWO-VGG16 + InceptionV3) using SPECT DaTscan dataset

A fully separate data subset that is previously prepared, is used to test and evaluate the effectiveness of the proposed models. Figures 7a-e and 8a-e show the confusion matrix for all the proposed models for both the datasets i.e. T1,T2-weighted MRI and SPECT DaTscan respectively.

#### Experimental results and discussions

Before obtaining the results of the proposed models, various preprocessing and hyperparameter optimization techniques are applied to obtain better accuracy and other performance measure results. Four deep learning models VGG16, DenseNet, DenseNet + LSTM, InceptionV3 and a hybrid model VGG16 + InceptionV3 are trained using the two standard datasets T1,T2-weighted and SPECT DaTscan with 9070 and 20,096 images. The results are briefly explained below for both datasets.

##### Results using T1,T2-weighted MRI dataset

The results evaluation and comparison of all the proposed models are presented in Table 7.

**Table 7:** Results of the proposed models using T1,T2-weighted dataset

Performance Measures	Proposed Models				
	**GWO-VGG16 (%)**	**GWO-DenseNet (%)**	**GWO-DenseNet + LSTM** **(%)**	**GWO-InceptionV3 (%)**	**Hybrid Model**
					**GWO-VGG16 + InceptionV3 (%)**
Accuracy	99.00	99.55	98.29	99.77	**99.94**
Sensitivity	98.20	99.44	96.27	100	100
Specificity	99.54	99.63	99.63	99.77	99.21
Precision	99.30	99.44	99.43	99.96	99.84
F1-Score	98.74	99.44	97.82	99.92	99.68
AUC-ROC	99.67	99.99	99.88	99.78	99.99
Training Loss	0.0272	0.009	0.575	0.0348	0.0272

The results of Table 7 demonstrate that all the proposed models achieved more than 99% of testing accuracy except GWO-DenseNet + LSTM which resulted in 98.29% accuracy and the hybrid model (GWO-VGG16 + InceptionV3) obtained highest accuracy 99.94% with the training loss of 0.0272 which is minimum among all models.

##### Results using SPECT DaTscan MRI dataset

The result evaluations and comparison of all the proposed models are given in Table 8.

**Table 8:** Results of the proposed models using SPECT DaTscan dataset

Performance Measures	Proposed Models				
	**GWO-VGG16 (%)**	**GWO-DenseNet (%)**	**GWO-DenseNet + LSTM** **(%)**	**GWO-InceptionV3 (%)**	**Hybrid Model**
					**GWO-VGG16 + InceptionV3 (%)**
Accuracy	99.77	99.52	99.75	99.71	**100**
Sensitivity	100	99.42	99.64	99.44	99.82
Specificity	99.21	99.65	99.85	99.68	100
Precision	99.84	99.72	99.90	99.69	99.99
F1-Score	99.68	99.66	99.87	99.45	100
AUC-ROC	99.99	99.52	99.73	99.71	99.92
Training Loss	0.0221	0.0033	0.0452	0.0412	0.0153

The aforementioned table demonstrates that all the proposed models achieved more than 99% testing accuracy. The hybrid model GWO-VGG16 + InceptionV3 obtained exactly 100% testing accuracy with the training loss values 0.0153 in comparison to other models.

The description of the validation of proposed models using independent data for both datasets is given in Table 9.

**Table 9:** MRI data sample distribution for independent dataset

Datasets Used	Total no. of subjects		Total no. of image samples used
T1, T2-weighted MRI images	**30**		**9086**
	PD	15	
	HC	15	
SPECT DaTscan	**36**		**20120**
	PD	18	
	HC	18	

##### Results using SPECT DaTscan MRI dataset

The results of Table 10 demonstrate that all the proposed models performed well and the hybrid model (GWO-VGG16 + InceptionV3) obtained highest accuracy of 99.54% with the training loss of 0.0027 which is minimum among all models.

**Table 10:** Validation of the proposed models using independent data of T1,T2-weighted dataset

Performance Measures	Proposed Models				
	**GWO-VGG16 (%)**	**GWO-DenseNet (%)**	**GWO-DenseNet + LSTM** **(%)**	**GWO-InceptionV3 (%)**	**Hybrid Model**
					**GWO-VGG16 + InceptionV3 (%)**
Accuracy	98.64	97.74	97.58	98.26	**99.54**
Sensitivity	97.21	97.98	97.25	98.36	99.14
Specificity	98.34	97.65	96.11	98.72	98.62
Precision	97.63	97.55	97.22	98.12	99.72
F1-Score	98.28	97.24	97.28	98.45	99.20
AUC-ROC	98.32	97.85	97.60	98.30	99.56
Training Loss	0.0027	0.009	0.0545	0.0034	0.0027

##### Results using SPECT DaTscan MRI dataset

Similarly, the above Table 11, results exhibit that all the proposed models performed well and the hybrid model (GWO-VGG16 + InceptionV3) resulted 99.11% accuracy during testing with the training loss values 0.0041 in comparison to other models.

**Table 11:** Validation of the proposed models using independent data of SPECT DaTscan dataset

Performance Measures	Proposed Models				
	**GWO-VGG16 (%)**	**GWO-DenseNet (%)**	**GWO-DenseNet + LSTM** **(%)**	**GWO-InceptionV3 (%)**	**Hybrid Model**
					**GWO-VGG16 + InceptionV3 (%)**
Accuracy	98.84	98.99	97.00	98.38	**99.11**
Sensitivity	98.09	97.87	97.41	96.79	95.00
Specificity	99.66	98.55	97.36	98.66	98.65
Precision	99.66	98.62	95.53	97.77	99.00
F1-Score	98.84	98.40	95.97	97.28	98.36
AUC-ROC	98.86	98.95	97.12	98.32	99.15
Training Loss	0.0045	0.005	0.038	0.0056	0.0041

### Comparison with the existing models

The proposed models' outcomes are presented in Tables 12 and 13 along with comparisons to other previously reported models. The comparison exhibits that for both datasets, the proposed deep learning models beat all other existing models in terms of performance metrics like accuracy, sensitivity, specificity, precision, f1-score and AUC score.

**Table 12:** Proposed models comparison with existing models using T1,T2-weighted dataset

Authors	Models used in their study	Accuracy (%)	Sensitivity (%)	Specificity (%)	Precision (%)	F1-score (%)	AUC-ROC score (%)
Lei et al. (2018) [41]	SVM	78.37	84.70	**-**	66.73	70.21	94.20
Esmaeilzadeh et al. (2018) [22]	Deep Learning	100	**-**	**-**	**-**	**-**	**-**
Shah et al. (2018) [23]	CNN	96	**-**	**-**	**-**	**-**	**-**
Ramamurthy et al. (2019) [42]	CNN	94.33	97.47	82.54	95.45	**-**	**-**
Mostafa et al. (2020) [20]	Ensemble Model	94.70	**-**	**-**	**-**	**-**	**-**
Sivaranjini, et al. (2020) [21]	CNN	88.90	**-**	**-**	**-**	**-**	**-**
Chakraborty et al. (2021) [18]	3D-CNN	95.29	94.3	94.30	92.7	93.6	98
Solana-Lavalle et al. (2021) [16]	Logistic, RF, NB, Bayesian Net, KNN, MLP and SVM	Men (99.01)	99.35	100	100	**-**	**-**
		Women (96.97)	100	96.15	97.22	**-**	**-**
Talai et al. (2021) [17]	SVM + MLP	95.1	-	100	-	100	**-**
Siddiqui et al. (2022) [43]	SVM	96.4					
Camacho et al. (2023) [14]	Explainable AI, CNN	79.3	77.7	81.3	80.2	-	87
**Proposed Model**	**GWO-VGG16 + InceptionV3**	**99.92**	**99.81**	**100**	**99.9**	**100**	**100**

**Table 13:** Proposed models’ comparison with the existing models using SPECT DaTscan dataset

Authors	Models used in their study	Accuracy (%)	Sensitivity (%)	Specificity (%)	Precision (%)	F1-score (%)	AUC-ROC score (%)
Martinez-Murcia et al. (2017) [31]	3D-CNN	95.5	96.2	**-**	**-**	**-**	**-**
Rumman et al. (2018) [30]	ANN	94	100	88	**-**	**-**	**-**
Kollia et al. (2019) [29]	CNN-RNN	98	**-**	**-**	**-**	**-**	**-**
Ortiz et al. (2019) [44]	CNN	95	95.5	94.8	**-**	**-**	97
Pianpanit et al. (2020) [26]	3D-CNN	96.87	97.10	97.89	**-**	**-**	-
Chien et al. (2020) [27]	ANN	99.22	81.8	88.6	**-**	**-**	-
Nalini et al. (2020) [28]	ANN	95	-	-	**-**	**-**	-
Mohammed et al. (2021) [25]	2D-CNN	99.34	99.04	99.63	**-**	**-**	**-**
Leung et al. (2021) [24]	CNN	**-**	**-**	**-**	**-**	**-**	84
Thakur et al. (2022) [11]	DenseNet121	99.2	99.2	99.4	**-**	99.1	99
**Proposed Model**	**GWO-VGG16 + InceptionV3**	**100**	**99.82**	**100**	**99.99**	**100**	**99.92**

The above table shows that, in terms of accuracy, from all the proposed deep learning models, hybrid model GWO-VGG16 + InceptionV3 outperforms the other eleven existing models and obtained 99.92% accuracy which is nearly similar to the model proposed by [22] with 100% accuracy.

Table 13 demonstrates that, in terms of accuracy, from all the proposed models, hybrid model GWO-VGG16 + InceptionV3 outperforms the other existing models with 100% accuracy.

When doing comparison on the basis of accuracy the reported machine learning models give the values as 99.01(Men), 96.97% (Women) [16], 85% [17], 96.4 [41] which is less than the result of the proposed deep learning models (99.94%) in T1,T2 weighted datasets. The papers [18] and [39] do comparison on the basis of AUC rather than accuracy and the values are 94.2% and 98% respectively. In the present investigation, the proposed deep learning models demonstrated an improvement of 1.9% to 4.8% in the AUC values in comparison to the existing models given in [18] and [39]. Similarly for the SPECT DaTscan dataset the proposed deep learning models exhibit better performance with 100% accuracy and 99.92% AUC in comparison to the reported literature [42] and [11] that give AUC of 97% and 99% respectively. Hence, in overall comparison, the proposed deep learning based models outperform the existing models in terms of accuracy and AUC values. The deep learning classification models presented in this study show the potential of such computational tools as future assistive diagnostic solution for doctors.

### Limitations of the study

The study has certain research limitations which are listed below :

- 1.To implement it needs high memory space more than 16GB RAM, a high-end GPU system in this case.
- 2.Because of the high-dimension data, it takes more time to execute and also increases the time and space complexity.
- 3.Only binary class classification problem is used for early prediction of Parkinson’s disease. Multiclass classification can also be done.

## Conclusion and future work

The detection of Parkinson's disease is becoming more and more crucial today. Because PD is a tremor illness, it is increasingly difficult to make an accurate diagnosis of the condition, especially in the early stages. This study proposes a classification approach for Parkinson's disease (PD) detection that enables doctors to make an accurate and timely diagnosis. The novelty of the paper is development of the hybrid model containing VGG16 and part of InceptionV3 whose hyperparameters are updated using GWO for PD detection which is first of its kind. The paper proposes four deep learning models, VGG16, DenseNet, DenseNet + LSTM, InceptionV3 and a hybrid model VGG16 + InceptionV3. In order to optimize the hyperparameters of proposed deep learning models, the GWO algorithm is used which automatically fine tune the hyperparameters and enhance the performance of the models. Two datasets T1, T2-weighted and SPECT DaTscan are used for the experiment. Various preprocessing techniques are applied to the images to enhance the models' functionality. After pre-processing, the GWO optimization algorithm is fitted into all the models and efficiently encoded. The hybrid model GWO-VGG16 + InceptionV3 has obtained 99.92% of accuracy and 99.99% AUC with T1,T2 dataset. While the same hybrid model has resulted 100% of accuracy and 99.92% AUC for SPECT DaTscan dataset. The paper also validates the effectiveness of the proposed hybrid model using independent data and demonstrated its superiority with accuracy and AUC values of 99.54% and 99.56% for T1,T2 weighted dataset and 99.11% accuracy with 99.15% AUC for SPECT DaTscan dataset respectively.

The work can be expanded in the future by adding new hyperparameter tuning techniques. Feature extraction will be done using Region of Interest (ROI) of two regions caudate and putamen in SPECT DaTscan dataset. Segmentation will be applied to the MRI images to detect the Parkinson’s disease. Also, work can be done on multiclass classification problems and more no of patients with larger MRI images.

The proposed model will help the physicians to detect the PD disease before the detection of motor symptoms which help them to initiate treatment timely and make proper strategy for better treatment and hence improved quality of their lives. Secondly, the proposed algorithm can be integrated with wearable sensors and devices for real time tracking of PD disease and hence can reduce the treatment time.

### Supplementary Information

Supplementary Material 1.

## Data Availability

The datasets generated and/or analysed during the current study are available in the PPMI repository, https://www.ppmi-info.org/.
