# Supplementary material for: An improved method for diagnosis of Parkinson’s disease using deep learning models enhanced with metaheuristic algorithm
Source: BMC Med Imaging. 2024 Jun 24;24:156. doi: 10.1186/s12880-024-01335-z (PMC11194992; doi:10.1186/s12880-024-01335-z)
Supplement: Supplementary file 1 — Supplementary Material 1. [file 12880_2024_1335_MOESM1_ESM.docx]

**Supplementary Figures**

| **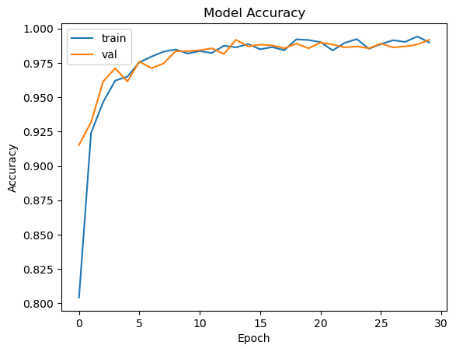** | **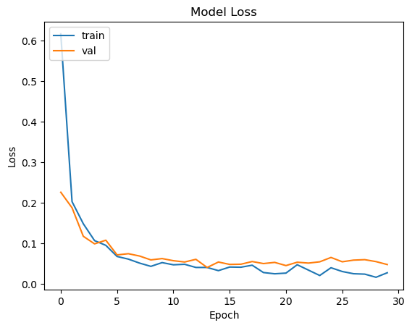** |
| --- | --- |
| **(a) GWO-VGG16** | |
| **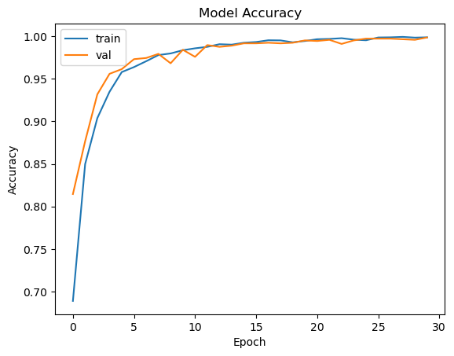** | **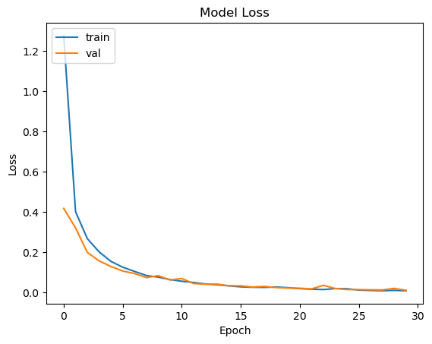** |
| **(b) GWO-DenseNet** | |
| **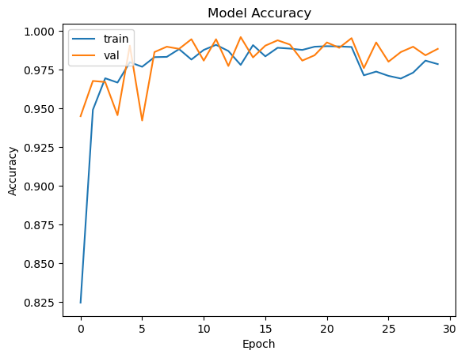** | **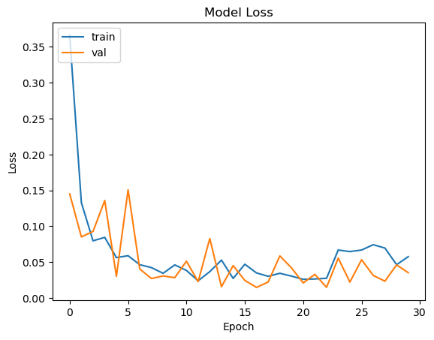** |
| **(c) GWO-DenseNet+LSTM** | |
| **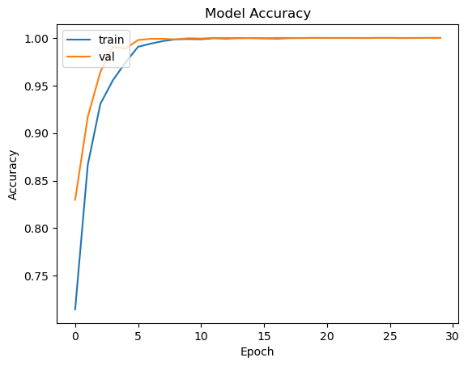** | **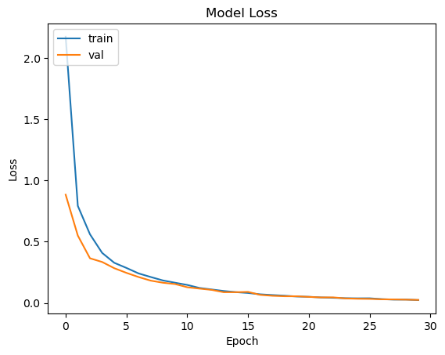** |
| **(d) GWO-Inception V3** | |
| **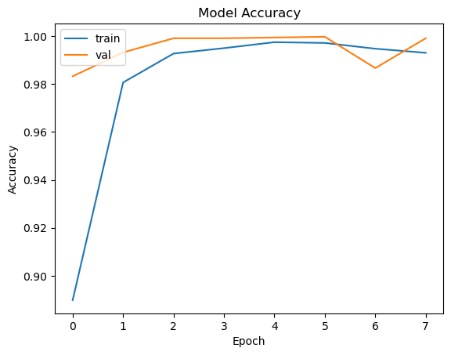** | **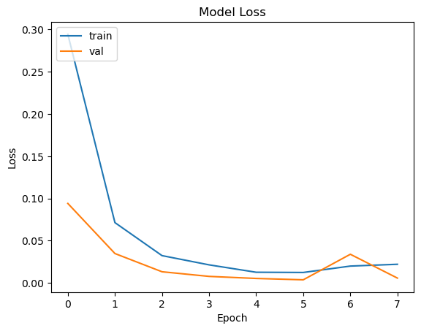** |
| **(e) GWO-VGG16+InceptionV3** | |

**Supplementary Fig. 1: Plots for accuracy/loss during training and validation of all the proposed models (a) GWO-VGG16, (b) GWO-DenseNet, (c) GWO-DenseNet-LSTM, (d) GWO-InceptionV3 and (e) Hybrid model (GWO-VGG16+InceptionV3) using T1,T2-weighted MRI dataset**

**Training/Validation accuracy/loss for SPECT DaTscan Dataset**

| **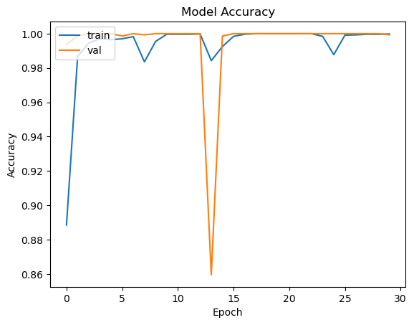** | **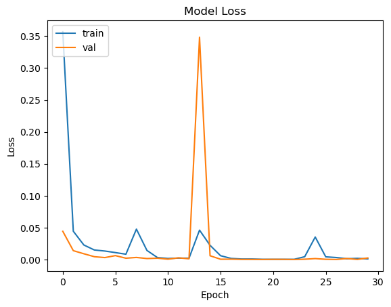** |
| --- | --- |
| **(a) GWO-VGG16** | |
| **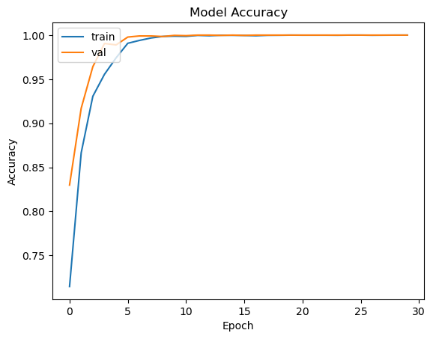** | **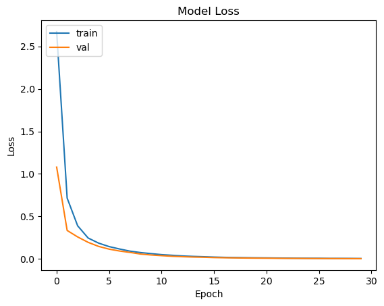** |
| **(b) GWO-DenseNet** | |
| **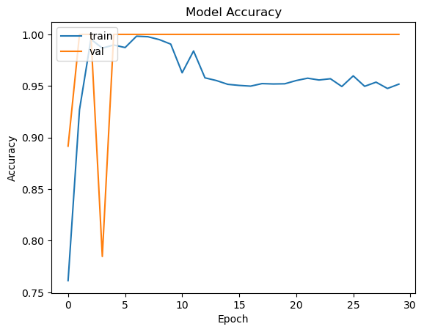** | **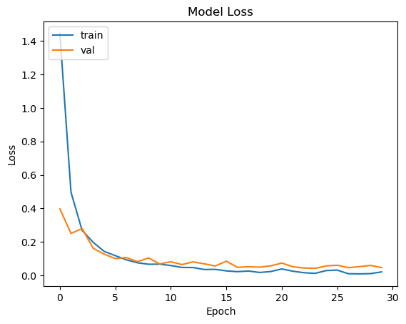** |
| **(c) GWO-DenseNet+LSTM** | |
| **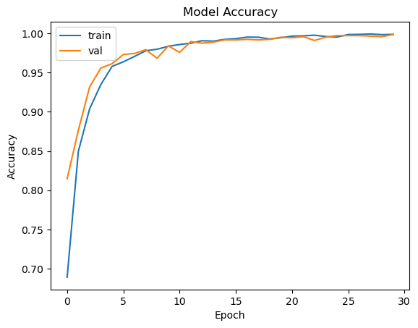** | **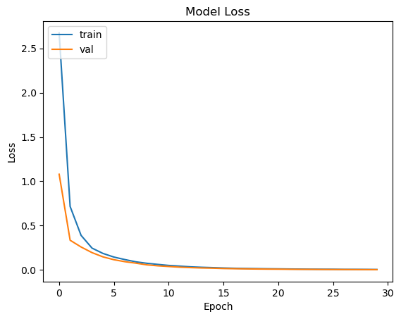** |
| **(d) GWO-Inception V3** | |
| **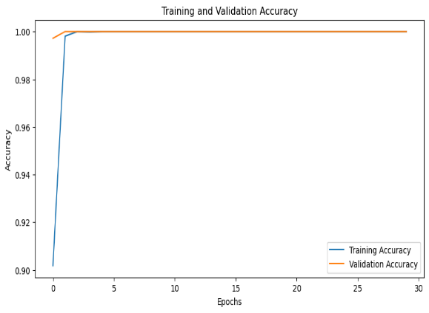** | **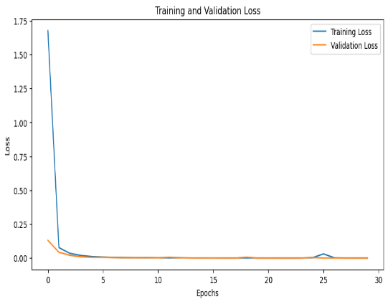** |
| **(e) GWO-VGG16+InceptionV3** | |

**Supplementary Fig. 2: Plots for accuracy/loss during training and validation of all the proposed models (a) GWO-VGG16, (b) GWO-DenseNet, (c) GWO-DenseNet-LSTM, (d) GWO-InceptionV3 and (e) Hybrid model (GWO-VGG16+InceptionV3) using SPECT DaTscan dataset**
